# An exploratory study on the perceptions of rabies and ill-health causations and health seeking behaviours of school children and local communities in southern Bhutan

**DOI:** 10.1186/s12889-023-15113-z

**Published:** 2023-02-07

**Authors:** Lungten Lungten, Tenzin Tenzin, Severine Thys, Waraphon Phimpraphai, Sangay Rinchen, Michel de Garine-Wichatitsky

**Affiliations:** 1 Regional Livestock Development Centre, Wangdue Phodrang, Bhutan; 2 World Organization for Animal Health, Sub Regional Represention for Southern Africa, Gaborone, Boswana Botswana; 3 ASTRE, University Montpellier, CIRAD, INRAE, Montpellier, France; 4 Faculty of Veterinary Medicine, Kasetsart University, Bangkok, Thailand; 5 Department of Livestock, National Centre of Animal Health, Thimphu, Bhutan

**Keywords:** Ill-health causation, Heath-Seeking behaviours, School Children, Communities, Rabies, Bhutan

## Abstract

**Background:**

The perception of illness and health-seeking behaviours, including rabies differ from one culture to another. Depending on the cultural setting of the society in which people live, the definition of the causal factors of illness may range from natural biological causes to supernational causes which greatly influence subsequent health-seeking behaviour. To ensure best health practices and plan effective health interventions for the control of dog-mediated-human rabies, we explored how school children and adult communities perceive and respond to illnesses, including rabies in southern Bhutan.

**Methods:**

We collected quantitative data related to the causes of illness and health-seeking practices of school children using a questionnaire survey (QS). Qualitative data were collected through focus group discussions (FGDs) and in-depth interviews (IDIs) with older members of communities (≥ 18 years) that work closely with school children. Descriptive analysis was performed for the quantitative data and thematic analysis was performed for the qualitative data.

**Results:**

The participants during the FDGsand IDIshave linked the illnesses to past actions, spirits, energy channels, planetary movement, sorcery, black magic, food, physical or environmental factors, individual habits and social factors. The survey of the school children reported microorganisms (75%), past actions (16.8%), spirits (9.6%) and black magic (9.1%) as causal factors for illnesses. Health seeking behaviours reported by the participants included visiting hospitals, performing spiritual or religious rituals and local treatments. Similarly, school children also mentioned that illnesses can be treated by visiting hospitals (98%), performing rituals (59.1%), and seeking traditional treatments practices (18.8%). Both school children and adult members of the communities that we interviewed were well aware on the causes of rabies and need for allopathic treatments rather than seeking spiritual or local treatments.

**Conclusion:**

There is a need for the consideration of the socio-cultural context in the planning and implementation of health-related policies, including the rabies prevention programs in Bhutan, by involving traditional healers and religious entities with “One Health” public health sectors.

## Background

The concept of “health” differs among different cultures which determine how societies develop their own medical systems [1]. The western biomedical explanation of ill health is primarily based on the biological theory which considers that diseases are the results of abnormal physical states caused by bacteria, virus, and hormonal or chemical imbalances in the body [2]. However, belief in supernatural causes like mystical causation (e.g., fate, ominous sensation), animistic causation (e.g., soul, spirit), and magical causations (e.g. sorcery, witchcraft) have been widely reported from many communities around the world [3]. Such reports are common in low and middle-income countries [4], in which a large proportion of people believe in the existence of supreme beings (e.g. Gods), supernatural agents (e.g. witches) ,or different kinds of spirits (e.g. natural spirits, ancestral spirits) that have the potential to put a spell on human beings and affect subsequent healing from illnesses [5]. For example, in Kenya, some communities believe that epilepsy is caused by natural spirits curses and ancestral spirits. They believe that specific rituals need to be performed to prevent the spirits of the deceased relatives from returning to their families and affecting future children with disorders [6]. Similarly, 53% percent of Malay people associate the causes of mental illness with supernatural agents such as witches and evil spirits and believe that these conditions can be treated by a traditional healer (*bomohs*) [7]. In these communities, the traditional practices not only help to complement modern biomedical treatments to produce the best individual health outcomes [8, 9], but it also supports countries to become self-sufficient in health care provision [10]. In recent years, an increasing number of countries have shown interest in their traditional practices [11]. The World Health Organization (WHO) also formally recognized and promoted traditional medicines as an indispensable part of health care systems in 1978 [10]. However, in a community where there are no proper regulations and awareness among the population, the practices could lead to serious consequences. Lots of deaths had been reported in many communities for failing or delaying medical treatments due to cultural beliefs and practices [12]. In cases of rabies, since washing of the wound and PEP were only proven effective methods [13], traditional remedies were considered as important risk factors [14, 15]. In Bhutan, people generally associate the occurrences of diseases or illnesses not only with physical or natural disturbances but also with several other non-medical factors such as mental and spiritual health, environment, economics, social and cultural milieu, and ethics or morality. To understand the health and diseases in their wholeness, all these conditional factors are to be taken into account. Bhutanese often believe that all the attributes of an individual, including his/her physical, mental, and moral dimensions, must be taken care of to restore their health. With such views and perspectives, approaches regarding the treatment of diseases are not only targeted for the corrections of physical causes through modern allopathic medicines or local herbal treatments, but also by addressing the psychosocial aspects of the disease. Thus, the mental well-being and health of ill persons are improved through the performance of diverse tantric anti-ghosts rituals and religious medicines [16]. For instance, in the hospital-based survey conducted by Pelzang in Bhutan [16]), 99% of the respondents reported that they would perform religious rituals/prayers when someone in the family is sick. Most people also report improvement of their health and well-being following traditional healing or religious practices or rituals [17]. Although the idea of a holistic framework is supported by many researchers [10, 11, 18], without proper knowledge and regulation of practitioners and clients, it may cause unnecessary delays in receiving biomedical treatments that could lead to worsened state or even death. It is specifically important for those infectious diseases like rabies, for which the identified biological cause must be treated in the hospitals only. In the current scenario, Bhutan does not have proper regulations and guidelines which can help to regulate traditional practitioners. Although some studies [16, 19, 20] have been conducted to understand the prevalence of local beliefs and practices, the targeted studies areas lack information on how local communities and key traditional practitioners perceive and respond to illnesses and their possible implications on rabies control programs. Therefore, in order to help strengthen future health intervention activities,we conducted an exploratory study to understand how local communities, school children and key traditional practitioners perceive and respond to illnesses including rabies.

## Materials and methods

### Ethic approval

The ethical approval for the study was obtained from the Research Ethics Board of Health, Ministry of Health (Ref. No. REBH/Approval/2019/113). Administrative approvals were obtained from the city education officers, school principals, and class teachers prior to school children questionnaire interview.

### Study area

**Fig. 1:**
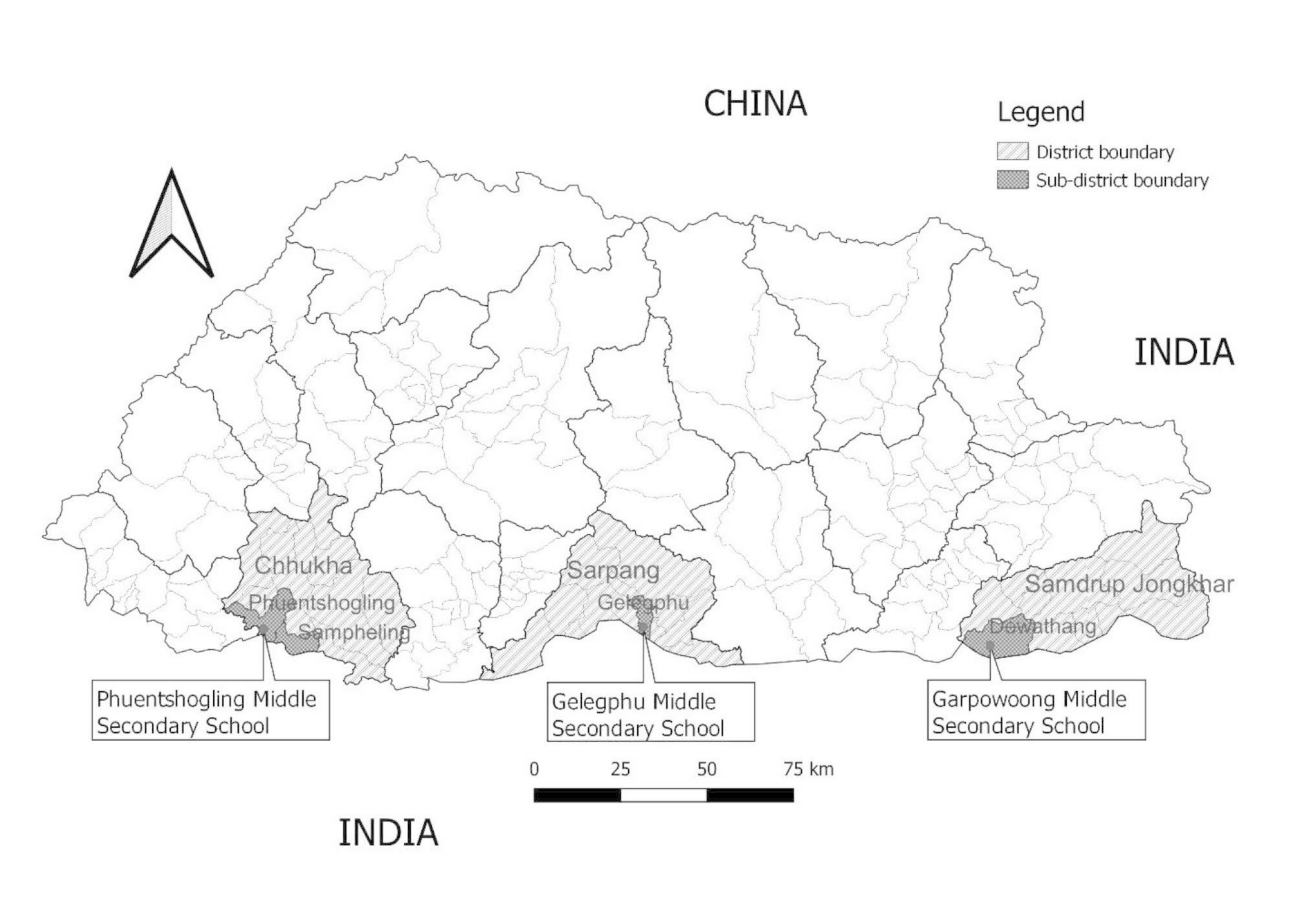
Map of districts, sub-districts and schools in Bhutan where the study was conducted. Map was drawn using Quantum GIS (QGIS Development Team, 2019)

The study was conducted in three areas of southern Bhutan - Phuntsholing, Samdrup Jongkhar, and Gelephu - where we previously conducted knowledge and perceptions studies about rabies among school children [21] (see Fig. 1). Cross-border dog-mediated rabies transmission is common in these areas, with reports of few human rabies deaths in the recent past [22, 23]. Previous studies had also identified rabies knowledge gaps among these communities [22, 24]) which might be occurring due to social and cultural factors. Therefore, our study was conducted to examine how school children and communities living in these three areas perceived and responded to illness and how such perception and practices can affect health intervention measures including rabies.

### Study participants and sampling

The study was conducted as part of a larger study looking at knowledge, attitude, and practices of school children on rabies and sociocultural factors that influence their perception and behaviours. The participants included both schoolchildren and adults. The school children participated in a structured questionnaire survey, while adults were included in Focus Group Discussions (FDGs) and In-depth Interviews (IDIs). For a survey, a total of 701 school children studying in class X in three middle secondary schools in southern Bhutan were conveniently chosen. In FGDs, those adults who closely interact with the school children (e.g., teachers, wardens, matrons, school children’s parents) and those having specific knowledge of culture (e.g. local leaders, astrologers, local healers) were randomly selected. Participants from different occupational groups that are involved in diseases and rabies control programs such as the Department of Livestock (DoL), Department of Forest and Park Services (DoFPS), Bhutan Agriculture and Food Regulatory Authority (BAFRA) were also included for FGDs. Twenty threee individuals who had participated in FGDs that were conducted in three groups - two on the school campus (Samdrup Jongkhar and Phuntsholing) and one outside of the school campus (Phuntsholing). Each FGD comprises of 7–8 participants and included both men and women. The key informants for the IDIs were chosen using snowball sampling method after the FGDs and consisted of local leaders, local healers, astrologers, Buddhist masters, business leaders, and farmers. A details description of the samples was given in Table 1.

**Table 1:** Socio-demographic characteristics of school children (n = 701) and associated adults who participated in the study (n = 23)

School children (n = 701)			FGD participants (n = 23)			KIs (n = 7)	
Variable	Freq (%)		Variable	Freq (%)		Variable	Freq (%)
Schools			Area			Area	
GMSS*	160(22.8)		PMSS	7 (30.4)		Phunstholing	4(57.1)
PMSS	304(43.4)		GaMSS	8(34.8)		Samdrup Jongkhar	3(42.9)
GaMSS	237(33.8)		CVH & SL	8(34.8)		**Age**	
**Age**			**Age**			Male	7(100.0)
<15 Years	463(66.1)		<34 years	15(65.2)		Female	0(0.0)
>15 years	238(34.0)		>34 years	8(34.8)		**Sex**	
**Sex**			**Sex**			<46 Years	3(42.9)
Male	295(42.1)		Male	14(60.9)		>46 years	4(57.1)
Female	406(57.9)		Female	9(39.1)		**Education level**	
**Grade**			**Education level**			None	3(42.9)
Grade VIII	154(22.0)		Secondary or less	6(26.1)		Secondary or less	1(14.3)
Grade IX	291(41.5)		Certificate/Diploma	6(26.1)		Degree	1(14.3)
Grade X	256(36.5)		Degree	10(43.5)		Buddhist Philosophy	2(28.6)
			Master	1(4.3)		**Occupation**	
			**Occupation**			Local leader	1(14.3)
			Teacher	9(39.1)		Buddhist master	1(14.3)
			Non-teaching staffs	6(26.1)		Astrologer	1(14.3)
			Livestock staffs	4(17.4)		Business	1(14.3)
			Regulatory staffs	1(4.3)		Traditional healer	1(14.3)
			Forestry staffs	1(4.3)		Livestock	1(14.3)
			Local leader	1(4.3)		Farmer	1(14.3)
			Care taker	1(4.3)			

### Data collection

Quantitative data from the school children were collected using structured questionnaires which were designed as part of a larger study conducted to understand the knowledge, attitudes, and practices on rabies, which has been published elsewhere [23]. Qualitative data were collected using FDGs and IDIs. To ensure standardization, all the discussions and interviews were guided by the standard set of questions. Probes and clarifications were sought as deemed necessary. During the process of discussions and interviews, the participants were asked to express themselves in the language in which they were most comfortable. Therefore, the sessions were run in English, Dzongkha (the national language), and Sharchop language (the native language of eastern Bhutan) interchangeably. Each FDG lasted for 2–3 h and one key interview (KI) took approximately 30 min to 1 h. All discussions and interviews were audiotaped and notes were also taken to allow cross-checking and accuracy of the data. Face-to-face interviews were conducted with all the KIs except for one KI which was conducted through a telephone call and social media network using Facebook messenger. Pictorial evidences of the local practices were also taken during the field visits for KIs.

### Data analysis

Descriptive analysis of the quantitative data collected from the school children was analyzed using R software [25]. The qualitative data collected during the FGDs and IDIs were analyzed using thematic analysis techniques [26] with the support of the Weft QDA freeware (www.pressure.to/qda). For qualitative analysis, narratives of IDIs and FGDs were transcribed into English, followed by coding into different themes and categories. Development and placement of the coded data under the relevant themes were performed by considering each line, phrase, or paragraph of the transcript. The transcripts were read carefully and cross-checked by listening to the digital voice records to ensure the accuracy of the data. The codes were refined, updated, or removed depending on the significance of the data. Colour shadings, pens, and papers were also used to assist in coding of the data. In each theme or sub-themes, the discussion and probes were conducted to establish the links from which the main conclusions could be drawn.

## Results

The results are presented in two broad themes -causation of ill-health and health-seeking behaviours following illnesses and potential exposure to rabies. The results from the survey of the school children were also placed under these two themes. Quotes and conversational trends are presented within inverted comma to support the themes. To maintain the original meaning of the respondents, some of the words were presented in the local language. However, wherever possible, the English translation has been provided in the text.

### Perception of the causes of illness and rabies

#### Karma (past actions)

Most of the participants linked the causes of illnesses to the “karmic effect”. A Buddhist teacher or Rinpoche (an honorific term used in the Tibetan language, literally meaning “precious one” and referring to those recognized as reincarnated, older, respected, notable, learned, and/or accomplished lamas or teachers of the Dharma) explained that “karma is the basis of all illnesses” which operates at a very subtle level.

He explained that –.

“Law of causes and effects does not change, which means bad actions follow bad results, and good action will follow good results. Karmic effects follow like a shadow all the time and never leave. If the illness is the results of ripening of karmic effects, such illnesses cannot be cured using the medicines or rituals”.

Once the karmic forces are ripe, there will be arising of the “84,000 obstructive forces” (nerig gye thri zhi tong- meaning 84,000 types of diseases) that can affect the functioning of the physical, mental and subtle body of human beings, which can weaken them and make the body susceptible to afflictions by various kind of illnesses. Another participant reiterated its importance by saying that “karma can affect the state of both the present and future life including the health status”.

**Fig. 2:**
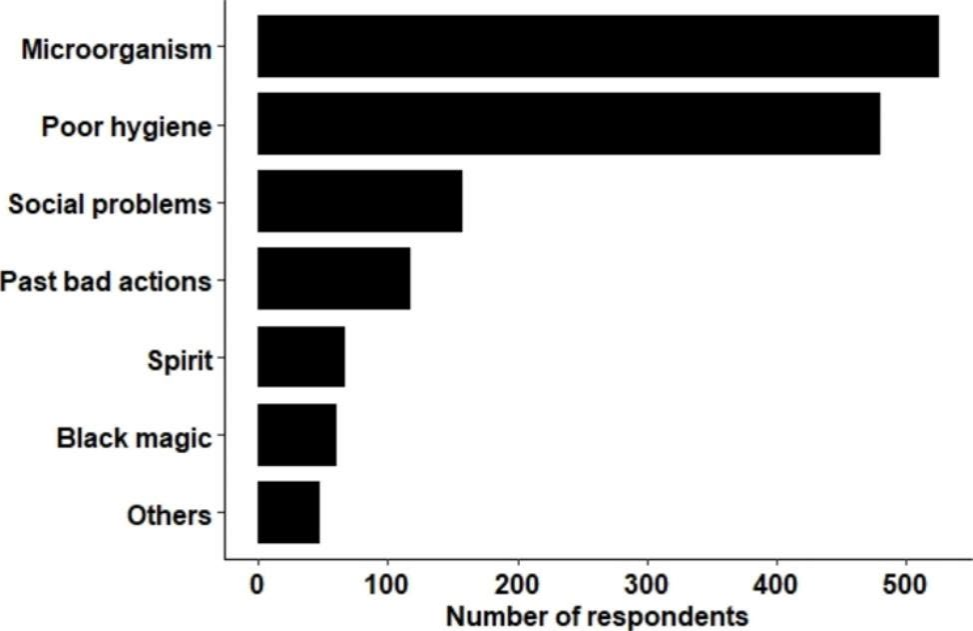
Causes of illness reported by school children in study area

The strong links between one’s actions with illness were also shared by the school children, with 16.9% reporting that illness can result from bad karma (bad actions committed in several previous life) (Fig. 2).

Since the links between illness and ‘karma’’ were found to be widely shared among the participants, we investigated whether our participants also linked the causes of rabies to “karma”. On enquiring about the possible causes of rabies, most of the participants understood that rabies occurs due to a bite from a rabid animal. For example, one of the respondents mentioned that rabies occurred due to a bite from a“mad dog”. However, most participants agreed that “karma” might create an enabling environment for the bites at the subtle level as one respondent said, “everything happens because of karma, including dog bites”.

#### Spirits and deities

Spirits and deities are considered another major cause of illness by most of the participants. Some of the names listed by the participants in local languages are “sadak” (Lord of the soil), “Neydak” (the guardian of sacred sites), “Lu” (water spirit /naga spirit that reside in water sources), “Sondre” (spirit of a living person), “Shindre” (spirit of a dead person), “Tshen” (local protective spirit), “Deuth” (spirit), “Chesung” (deity entrusted with protection of dharma), “Kasung”(deity entrusted with the protection of dharma by command), “Mamo” (wrathful feminine deities), “Rongthung abi memi” (spirit from Rongthung village in eastern Bhutan), “Phoshing” (male spirit), “Moshing” (female spirit) and “Neypo” (owner of a place owned by the spirit). A local astrologer said, “The pain in the body is not necessarily caused by diseases. Some are afflicted by Shindre, some by Tshen, some by Sadak and Lu”. One of the farmers mentioned the spirit’s name in local language as “Mema yin” (the spirit that are formless and not visible to human eyes). “Mema yin is like the wind. We cannot see them, but it afflicts us just by thinking about it”. An astrologer believed that some of the spirits are wandering “consciousness” of dead animals or humans suffering in the intermediate state (life between birth and death, called “Bardo”). Some spirits are guardians of elements like fire, water, tree, and air (e.g Lu, Sadak, Neypo), while others are deities or dharma (religious) protector (e.g., Chesung, Kasung, Nyedak etc). He explained that spirits can affect human beings for different reasons. For instance, when they need food and water or when they need the dedications of good deeds for them (i.e. Shindre), or when human beings disturb their surroundings (e.g. Sadak, Lu). One of the KIs explained that:

“When our parents or grandparents die, they expect offerings and afflict the living people when they are not offered. For example, if a dead person is a good friend of us or a family member, their soul would expect close people to offer and dedicate good things for them when they are suffering in the intermediate state after death - Bardo”.

The spirits can also affect the animals as reported by one respondent as-.

“Yes sure, animals will be also affected. When dog, cow, or horse are sick and if doctors don’t find any diseases, they might be affected by the phosing or moshing”.

From the survey we carried out in three schools, 9.6% mentioned that illness can be caused by the spirits (see Fig. 2).

#### Energy channels and planetary movement

Most participants believed that disturbance in energy channels inside our body can result in illnesses. A spiritual teacher and a local astrologer have explained that there are several energy channels in our body through which *rlung* (plainly translated as “life energy”) flows and maintains our life. The teacher said, “there are 3 major channels and 21,000 minor channels” in our body. Any change in the physical environment, body function, and different levels of mental emotions have the ability to affect the flow of *rlung* inside the body whichcan ultimately result in different types of illnesses.

The link between illnesses and planetary alignments was shared by some of the participants. They shared that everything in the universe is “interdependent” and any changes in one thing have abilities to affect others. The astrologer explained that there is a period in the life of human beings when the body is not in harmony with the planetary alignments which can result in illnesses. Some of the examples of such periods, as mentioned by astrologers, are called “Chak”, “Loka” and “Cho” in the Buddhist language.

#### Food

Food as the cause of illnesses was reported by most of the participants. They mentioned that consuming of “poor quality” and “unhygienic ” food can result in illnesses. Since food is essential for the body to “function normally”, they reported that the food consumed by people needs to be clean and fresh. One representative participating in an FGD explained that “spoiled food can be contaminated with lots of microorganisms which can damage our body”. Moreover, a few of the participants reported that food animals also need to be reared in a clean environment to prevent humans from contracting diseases. One participant felt that imported meats are unhygienic and not fit for human consumption when he said -

“The people along the borders let the pigs freely scavenge without any care. Pigs can feed on human feces/stool and on all types of waste materials”..

Some of the KIs participants considered that chemicals in vegetables and fruits are likely causes of various illnesses. They strongly felt that the vegetables that are purchased from outside the country contain high levels of chemicals.

#### Physical or environmental factors

Changing in the weather patterns, dirty surroundings and destructions of wildlife habitats due to developmental activities were cited as major physical or environmental factors leading to causes of illnesses in people. All participants mentioned that dirty surroundings attract lots of “microorganisms” and “flies” which then transmit many diseases to human beings, resulting in illnesses. When comparing if the school children from the survey also have similar perceptions, 75% of them reported that microorganisms are responsible for causing illness and 68% of them linking illnesses to poor hygiene and sanitation.

Some participants explained that due to developmental activities, the habitat of wild animals was destroyed at unprecedented rates, which brings wild animals closer to human dwellings and transmit diseases.

Due to developments, we destroy the habitat of wild animals. Wherever there is forest, we construct the houses. As a result, we can see now that wild animals are getting closer to human beings. For example, many monkeys have started to come inside our home. Similarly, wherever we go, there are rats in people’s home. People are not being careful. When we sleep, the rats get into the kitchen and eat the food if the lids of the pots are not closed, and there is a risk of disease transmission to humans

Another participant cautioned on immediate need for some interventions when he said:

“If wild animals are not taken care at present, there are chances that they can transmit many diseases to humans”.

Moreover, increases in the incidence of tick-borne diseases were reported by some participants due to the destruction of environments. When probed whether he had seen any cases of tick-borne infection, one farmer explained that -

“I have seen two people from the village coming to hospital complaining of fever, headache and giddiness after being bitten by ticks”..

Some believed that illnesses can be due to “seasonal changes”. For example, “dengue is caused when season changes and when there is lot of mosquitoes” said one respondent.

#### Individual/social factors

Drinking alcohol, lack of physical exercise, not wearing a facemask, and family problems were reported as some of the important factors responsible for illnesses. “Liver damage” and “Non-Communicable Diseases (NCD)” were mentioned as examples of illnesses by the participants that can results from the consumption of alcohol and lack of exercise respectively. The importance of wearing a facemask to prevent illnesses resulting from diseases like “COVID-19” was shared by most of the participants. Few of the participants felt that wearing the mask should be taken up as “individual responsibility” rather than being enforced by law by the government. Family problems such as divorces and fighting between parents were mostly mentioned by the teachers as one of the important causes of ill health in school children. Similarly, 22.5% of the school children also linked illnesses to social problems including divorce, fighting, and economic hardships.

#### Sorcery and black magic

Few respondents reported that illnesses can also result from sorcery and black magic. Although none of them were able to explain how it was done and how it affects people’s health, most of them agreed that it’s done when people have a “conflict with each other’s”. 9% of the school children surveyed also felt that illnesses can result from sorcery and black magic (see Fig. 2).

### Health seeking behaviours

The participants in our survey reported various forms of health-seeking behaviours following an illness. The behaviours and practices were reported under the following sub-themes:-.

#### Modern allopathic treatments

Seeking medical care from hospitals was the first preference for all participants following illnesses. Irrespective of the causes, all agreed that they would visit hospitals if they, or their family members, are ill. Most of them noted that “allopathic treatments” and “religious/traditional healing” should be used in complementarity to each other and believed that combining these two methods was the “best approach for the treatment of any illness”. The hospital treatments were mostly preferred when they felt that the causes are physical such as food and change in weather conditions.

**Fig. 3:**
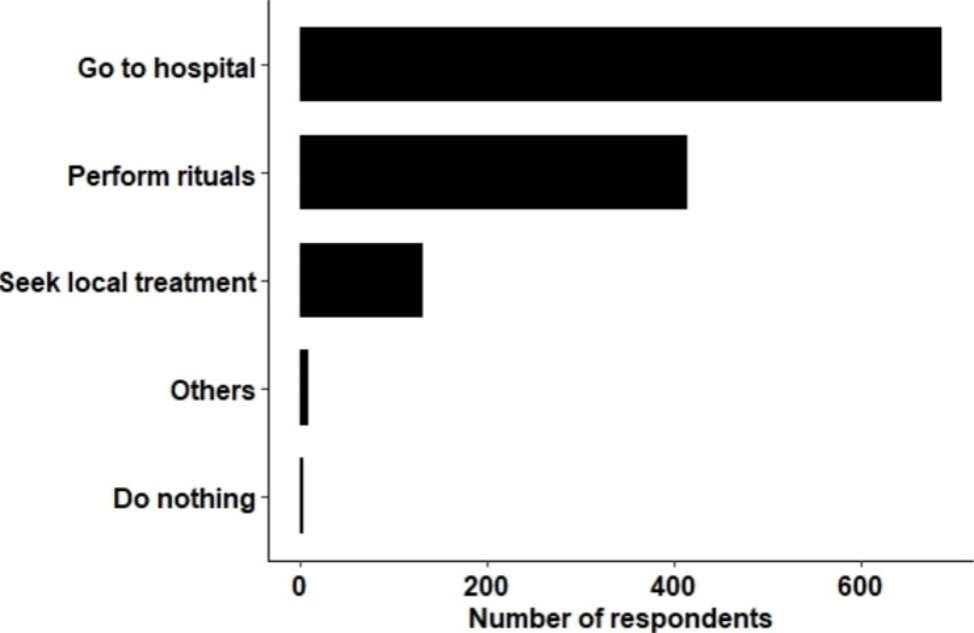
Health seeking behaviours reported by school children in study area

When the causal factors are suspected to be the causes of illness such as “karma” and “spirit”, biomedicines would be of little help, reported as having “little effect” by one of the participants. On exploring the behaviour and practice of the school children, almost all school children (98%) reported that they will visit the hospital for treatment (see Fig. 3).

#### Religious healing and rituals

Although hospital treatment was the most preferred treatment by most of the participants following illnesses, they agreed that seeking guidance from spiritual persons was appropriate, particularly for those illnesses that are chronic and can not be treated by modern hospitals. Similarly, more than half of the school children from three survey schools agreed that they would perform rituals in their house whenever they are ill (see Fig. 3). The Buddhist master explained that such illnesses usually result from the “ripening of karmic effects” and added that “taking such patient to the hospital for treatment will have little effects”. He advised -.

“Cleaning of the demerits and accumulating the merits is the only way of getting out of such diseases. If ample accumulation of merit is done, there are chances of getting out of the illness, but some can remain as a lifelong illness. Once the demerits are cleaned, medical and rituals will help to cure the illnesses”.

Most participants from FGDs and IDIs believed that “rituals would help in improving the overall outcome of ill patients” and agreed that they would even consider performing rituals if their family members are ill due to a dog bite. They believed that performing rituals would help to “clean the karmic effect and brings in the favorable circumstances”.

The participants in our survey came up with the names of numerous religious rituals. Commonly mentioned rituals or practices that can help to recover from illnesses included: “Kago” (recitation of prayers and mantras by lamas or rinpoche to the sick person), “Jabthri” (cleaning with blessed holy water performed by lamas (is a title for a teacher of the Dharma in Tibetan Buddhism), recitation of “Sangay menla” (medicine buddha) and “Vajrasattva mantra” (one of Buddhist deity) mantra, and burning of “Duzey” (similar to mustard grain size pills blessed by rinpoches and believed to cure illnesses), and “Dribsang” (burning of the dried powder from a tree believed to remove the material or moral pollution known as ‘drib’). For a person suspected of being affected by spirits, local astrologers and healers recommended burning butter lamps and “Sur” (burning of maize/wheat flour). They believed that “if we offer Sur or butter lamp, it will help them”. Few participants believed that hungry ghost spirits can be satisfied by the smell of the Sur offering. Since local astrologers and healers were actively involved in treating illnesses in the community, we sought to understand how they would treat rabies patients. On enquiring, if they had handled any rabies cases during their career, both KIs reported that they had not treated any rabies suspect patients. However, they said that “few patients with Central Nervous System signs” visited them, which would be difficult to treat them, although they indicated that religious rituals would be recommended for a person with such signs. On asking if they would be able to treat rabies patients, both agreed that they would not be able to cure such illness and recommended allopathic medicines as the best option. However, they also strongly believed that “rituals would help improve the outcomes”.

#### Local treatments and practices

In addition to modern medical treatment in hospitals and spiritual healings, local medications practices were reported in our study area. 18% of the school children that we survey also reported that they seek local treatment to cure their illness (see Fig. 3). Most of the services for local treatments were provided by local healers. One healer claimed that he had treated “more than 1000 patients” in the same locality in which he was living. Most of the patients that he treated were “those suffering from headaches, giddiness, stomach pain, and back aches”. Depending on the nature of the diseases, the local healer provided different treatment regimens like medicinal herbs, acupuncture, steam bath, hot iron plates, and mantra recitations. He reported that medicinal plants for his patients were collected from many different locations and are given both for internal and external use. All the knowledge and skills for diagnosis and treatments were claimed to be learned from ancestors who in turns studied standard Buddhist texts. During our study, we observed a traditional healer using a hot iron plate and ‘*vajra’* (‘indestructible thunderbolt instrument’) to treat a patient complaining of leg pain. With this method, the healer invoked the mantra using a rosary, blew on the painful area of the patient’s body, rubbed the area with mustard oil, heated the area with the hot iron plate, and pointed the *vajra* towards the painful area. He reported that such methods are performed for “illnesses like headaches, pains in legs and hands, and high blood pressure” and mentioned that it should not be used to treat “children and pregnant women”, as it could cause abortion. When we requested a local healer to share some examples of his treatment success, a local healer gave the example of a girl who came to him with a swollen leg. He explained-

“Once, there was a girl at Therbam village with a swollen leg. She was taken to the hospital and was given an injection. Blood was also taken from her leg but was not responsive to its treatment. I collected the medicinal leaves from forests, heated it, and applied a paste on her leg, and she was cured”..

In addition to the local medication practiced by the local healers, a few participants from KIs and FGDs also reported some local practices that they performed when they are ill. For example, one participant from FDGs reported that they drank juice from papaya leaves to cure dengue.

“Drinking the juice of papaya leaves (*Carica papaya*) mixed with coconut juice would help in curing the disease in human beings, and it was practiced by people during the 2019 dengue outbreak in Phuntsholing”.

Other participants talked about the effectiveness of “dried bile of an Asian black bear (*Ursus thibetanus*)” for treating various illnesses. On probing whether participants had performed or will performed any kind of local treatments to the dog bite wound, all respondents were not in favour of performing local treatment of dog bite wound. However, one farmer from the IDIs had reported that he had heard that dog bite victims can be treated by applying clean soil blessed by mantra from local healers after thorough washing.

“After thoroughly washing the wound, get soil from a clean area, roll it in the hand, blessed it with the mantra from local healers, and put it on the bite sites. That will also cure the wound”.

We also explored the common practices that were performed by schoolchildren in the study area. Almost 19% of the students reported that they will seek local treatment to recover from illnesses. Unlike local healers, the remedy reported by other KIs were found to practice through trial-and-error methods or through advice from friends/relatives/neighbors who are not standard practitioners.

## Discussion

In this study, we explored how school children and adult members of communities in the areas surrounding schools in three small towns in Southern Bhutan perceive and respond to illness and rabies. In line with the calls by WHO and several scholars, we adopted a holistic approach considering the multiple facets of the overall health and well-being of people [16, 17].

Our study revealed that there are huge influences of core cultural values on how the communities define and respond to illnesses. The influences were not only limited to adult members of the communities but were found to be extended to young school children. They define illnesses not only from biomedical perspectives, but their definition includes complex interactions between physical, social, and supernatural origins. Most of the participants, including school children, mentioned past actions, spirits, sorcery, energy channels, and black magic as some of the causal factors for illnesses. The anecdotes of such religious or supernatural entities responsible for causing illnesses in living beings can be abundantly found in many Buddhist texts [27, 28]). According to the twelfth-century Buddhist text entitled ‘four treatises or four tantras’ (*rGyud bzhi*), the “karmic force” which is mentioned as one of the important causal factors by many participants including school children is linked to three mental poisons (attachment or desire, aversion or hatred, and indifferences or delusion) and three humours (*rlung* /wind, *mkrisp*a/bile, and *bad kan*/phlegm) [28]. It is said that the force from past actions can create disturbance in the functioning of the three mental poisons and three humours, giving rise to a variety of illnesses. In addition to complex views of the illness, our study also revealed that most participants actively take part in religious rituals and local treatments following illnesses. Most of them, including 50.1% of school children, agreed that they would perform rituals whenever theyor their family members were ill. Irrespective of the causes of the illnesses, they believed that rituals would help to “improve the outcomes” of illnesses. Moreover, many of the participants mentioned local treatment as an alternative mode of treatment for recovery from illnesses. Given the fact that a large number of people are keen on performing religious rituals and traditional treatment, it is important to bring together all the practitioners of local or religious healing and health professionals to work collaboratively under a common platform. Engaging them collaboratively has the potential of not only improving the overall outcomes and well-being of the communities as, emphasized by WHO [10] and many experts [5, 11, 29, 30], but it will also help to preserve the intangible traditional heritage of Bhutan. The attempts have been made by governments through the integration of traditional healing practices known as “sogwa rigpa” into the mainstream allopathic hospitals in Bhutan [31]. But under the current scenario, there are no proper legal frameworks and policies that were established to guide local healers during their services. In absence of proper training, certification, and regulation by the government, wrong treatments and guidance can happen, which can put the client’s life at risk. Although such instances have not been reported in our study, with most participants supporting allopathic treatment as the primary choice and traditional practices as complementary measures to treat dog bites and prevent rabies, many reports indicated that cultural beliefs and practices could delay or even prevent people from availing the medical services [12]. Therefore, awareness programs forschool children and communities, and information-sharing platforms between health professionals and local practitioners are urgently needed. As emphasized by most key informants, there is also the need for further research to explore the ethnomedical practices which could complement the allopathic health care system. Few studies conducted in Bhutan have found that local or traditional healers have good knowledge of ethnomedicinal plants and home remedies which needed to be capitalized [32, 33].

In terms of rabies, our findings indicated that most of the participants understand that rabies is due to biological causes and not as a result of any supernatural or religious factors. They also understood that rabies can be treated only through washing animal bite wounds with soap and post-exposure prophylaxis, with some participants referring to it as “very necessary”. The local spiritual healers we interviewed confirmed that if they receive any patients with dog bites or suspected rabies cases, they would “immediately refer to hospitals” rather than treating the patients with the local healing methods. The finding indicated that there is a high awareness level of rabies in Bhutanese communities. The high level of knowledge on rabies among Bhutanese communities and school children was reported in a few other studies [21, 24, 34]. However, our findings on strong influences of social and cultural values in the way of how people perceive and respond to illness need to considered as one of the risk factors for rabies. Due to the presence of a large number of free roaming dogs and reports of high incidences of dog bites in the country (7000 bite cases/year) [22], a slight misinformation can be fatal for the victims. Therefore, awareness programs are needed to carried out regularly and all relevant stakeholders including local or spiritual healers should be actively engaged in rabies prevention and control program.

## Limitations

Because of COVID-19 pandemic restrictions, schools had to be chosen conveniently depending the approval from the government and school administration. Due to same reason, we were also unable to engage some of the key stakeholders (e.g., health) in FGDs and KIs as initially planned, which could have added more values to our study.

## Conclusion

Social and cultural values strongly influence the perceptions and health-seeking behaviour of school children and adult communities in our study area. Their definition of illnesses and subsequent behaviours are not only limited to the biomedical approach but extend to include complex interactions between physical, psychological, and supernatural models. Tostrengthen the health care system, and improve overall health outcomes and well-being of the people, efforts are needed to engage all relevant stakeholders including local or spiritual healers in health intervention measures, including a rabies control program.

## Data Availability

The quantitative datasets generated and/or analyzed during the current study are available in the [Zenodo] repository, [https://zenodo.org/record/5807315]. However, qualitative datasets generated and/or analyzed during the current study are not publicly available due [to the presence of confidential information and agreements made with key informants during the time of data collection but are available from the corresponding author on reasonable request.

## Abbreviations

BAFRA: Bhutan Agriculture and Food Regulatory Authority
COVID-19: Coronavirus disease 2019
DoFPS: Department of Forest and Park Services
DoL: Department of Livestock
FDGs: Focus Group Discussion
IDIs: In-Depth Interviews
KIs: Key Interviews
NCDs: Non-Communicable Diseases
QS: Questionnaires survey
WHO: World Health Organization
